# The World Health Organization Quality of Life instrument for people with intellectual and physical disabilities (WHOQOL-Dis): evidence of validity of the Brazilian version

**DOI:** 10.1186/1471-2458-14-538

**Published:** 2014-05-30

**Authors:** Juliana Bredemeier, Gabriela Peretti Wagner, Marilyn Agranonik, Tatiana Spalding Perez, Marcelo P Fleck

**Affiliations:** 1 Hospital de Clínicas de Porto Alegre (HCPA), Porto Alegre, Brazil; 2 Universidade Federal do Rio Grande do Sul (UFRGS), Porto Alegre, Brazil; 3 Faculdade de Desenvolvimento do Rio Grande do Sul (FADERGS), Porto Alegre, Brazil; 4 Universidade Federal de Ciências da Saúde de Porto Alegre (UFCSPA), Porto Alegre, Brazil; 5 Universidade do Vale do Sinos (UNISINOS), São Leopoldo, Brazil

**Keywords:** Quality of life, Disabilities, WHO, Measurement scale development, Scale validity

## Abstract

**Background:**

The number of people with disabilities in Brazil and worldwide has grown substantially in recent decades. Cross-cultural quality of life instruments can be helpful in the development of interventions designed to meet the needs of this population and contribute to rational allocation of resources. This study sought to provide evidence of validity and reliability the Brazilian Portuguese version of WHOQOL-Dis-D (a cross-cultural, multicentre instrument developed by the WHOQOL-Group for the assessment of quality of life in persons with physical disability – PD) and WHOQOL-Dis-ID (for persons with intellectual disability – ID).

**Methods:**

Classical psychometric methods were used to conduct independent analyses of the PD and ID samples. Criterion groups were established for analysis of construct validity. Concurrent validity was assessed in relation to SWLS and BDI-II scores; discriminant validity, in relation to WHODAS-II. Cronbach alpha was used to test the instrument scales and subscales for reliability. The ID subgroup was retested, and test-retest reliability assessed by means of intraclass correlation coefficients and paired Student’s *t*-test.

**Results:**

A total of 162 (98 females) people with PD and 156 (55 females) people with ID participated in the study. Cronbach alpha was satisfactory across practically all domains and factors in the PD subsample. In IDs, most factors or domains had coefficients higher than 0.70, but four subscales exhibited less satisfactory performance. Evidence of construct and concurrent validity and reliability were obtained.

**Conclusions:**

The analyses presented herein provide satisfactory evidence of the validity and reliability of the instrument and corroborated the factor structure revealed during cross-cultural research. Further studies with larger sample sizes are required to obtain additional evidence of validity and reliability.

## Background

Disability is an umbrella term used by the World Health Organization (WHO) to define impairments, activity limitations, and participation restrictions caused by a health condition [1], whether an impairment or a chronic illness. The number of people with disabilities has grown with each passing year. It is estimated that there are over 1 billion disabled persons worldwide, with approximately 200 million of them experiencing very significant difficulties [2]. This growth has been driven both by the increasing life expectancy and by an exposure to factors such as traffic accidents, urban violence, warfare, stress, drug misuse, HIV/AIDS, and malnutrition. Due to these and other factors, it is estimated that an individual born in a country where the mean life expectancy is 70 years of age will spend, on average, 11 years of his/her life with some form of disability [2]. In 2000, persons with disabilities were estimated to account for 14.5% of the Brazilian population [3], whereas in 2010 they represented nearly 22% of the population [4]. Even though this percentage may be overestimated due to possible problems in the Census data collection, the number of people with disabilities is growing as population is shifting toward an inverted age structure — a phenomenon already observed in Europe that means that over the following decades the number of people over the age of 40 will exceed that of younger individuals [4].

This context has generated growing research interest into the living conditions of persons with disabilities and their opinions on a variety of aspects, and has prompted the development of public policies to support health, well-being, and inclusion. This focus is consistent not only with the concept of health adopted by WHO [5], but also with the biopsychosocial model advocated by WHO within the framework of the International Classification of Functioning, Disability and Health (ICF) [1]. According to the ICF, disability is the opposite of functionality, both of which being the result of changes in the body and its consequence in the activities and participation of people. Thus, problems relating to activity limitation or participation restriction are described as disabilities. On the other hand, non-problematic aspects of health and states related to it are defined under the term functionality. In other words, the term disability is used to “to denote a multidimensional phenomenon resulting from the interaction between people and their physical and social environment” [1]. Impairments are defined as “problems in body function or structure as a significant deviation or loss” [1]. In Brazil, where the term disability is considered pejorative (such as if it referred to a lack of ability), there was a movement to instill a preference for the expression *person with special needs* to designate those people with an injury or disability. Historically, the term impairment has been used interchangeably with this expression within this country. However, neither the term impairment nor the expression person with special needs fill in the gap between the presence of an injury or disease and the occurrence of a loss of functionality. This is due to the fact that they do not take into account the fact that “two persons with the same disease can have different levels of functioning, and two persons with the same level of functioning do not necessarily have the same health condition” [1]. The matter of the term disability not being one widely spread in Brazil had an impact on data collection which we explain further.

This biopsychosocial approach of ICF has been recently reinforced by the work of authors such as Albrecht e Devlieger, who found that even people with moderate to severe disabilities often report good or even excellent quality of life (QoL) [6]. There are many definitions in literature for QoL, with the one published by the WHO in 1995 being on the most accepted. According to the WHO, QoL refers to “an individual’s perception of their position in life in the context of the culture and value system in which they live and in relation to their goals, expectations, standards, and concerns” [7]. QoL assessments can play a role in the development of medical and psychological interventions with greater sensitivity and effectiveness and are of major importance in the planning and follow-up of pharmacotherapeutic interventions and patient recovery [8-10]. Overall, the advent of instruments for measurement of QoL in different populations has also been an efficient tool for the assessment of potential stressors of human development and for the identification of the various predictors of QoL across diverse cultures. Furthermore, effective measures for the assessment of QoL also allow for the evaluation of social programs designed to improve QoL in specific populations or in the general population [8,9]. Some authors have argued that the inclusion of QoL measures in studies of new medical treatments outcomes can provide information to assist decision-making in resource allocation [11].

The search for a cross-cultural instrument for the assessment of QoL in people with physical and intellectual disabilities (PD and ID) led the WHO to conduct the project called “Quality of Care and Quality of Life for People with Intellectual and Physical Disabilities: Integrated Living, Social Inclusion and Service User Participation Project”, also known as the DISQOL project. This program, in turn, led to the development of instruments for the assessment of QoL, quality of care, and attitudes toward disabilities (unpublished observations). The cross-cultural development of WHOQOL-Dis was described in detail by Power, Green, and the WHOQOL-DIS Group [12]. The results of concept exploration by means of focus groups in Brazil were published elsewhere [13].

The development of the Brazilian-Portuguese versions of WHOQOL-Dis for participants with PD and ID was led by the Brazilian branch of the WHOQOL-Group. The primary objective of this study is to present the Brazilian Portuguese version of WHOQOL-Dis and provide evidence of its validity in and reliability. Our specific objectives were to provide evidence of (1) construct validity, by means of factor analysis, internal consistency (Cronbach alpha), and hypothesis testing (criterion groups), (2) criterion validity, through concurrent and discriminant validity, and (3) test-retest reliability.

## Methods

The WHOQOL-Dis items are expected to be administered jointly with WHOQOL-Bref, a cross-cultural 26-item generic instrument for the assessment of generic QoL [14]. Therefore, WHOQOL-Dis actually consists of the administration of WHOQOL-Bref with the addition of the DISQOL module.

Concept exploration with focus groups led to the development of a pilot version of the DISQOL module [12]. The final version was then qualitatively analyzed by the Brazilian research group and compared against the topics generated by the focus groups round. Three Brazilian judges experienced in the matter of disabilities confronted each of the original English-language items with the categories resulting from the content analysis of focus group discussion transcripts. A local item was created whenever a category identified on content analysis was not represented among the original items and when at least two judges believed its inclusion would be essential to portrayal of the reality of persons with disabilities in Brazil. This analysis led to the creation of five local items: “environmental adaptations to limitations”, “physical barriers”, “job opportunities”, “study opportunities”, and “feeding”. A detailed rationale for keeping or excluding items after the pilot study stage is provided elsewhere [15].

Psychometric analyses of data obtained by the 15 participating centres of the pilot study were carried out at the coordinating centre [12]. The results of this analysis suggested a need for development of a cognitively simpler instrument for participants with ID, and that the use of a three-point rather than a five-point Likert scale would improve the psychometric performance of the instrument in this sample [16]. For participants with PD a five-point scale was maintained.

The following instruments were used in the research protocol:

1. WHODAS-II: a measure of disability developed by WHO [17] within the ICF framework [1]. The 12-item version is available both as a self-administered questionnaire as an interviewer-administered instrument. Cronbach alpha: 0.98. As no Brazilian Portuguese version of WHODAS-II was available at the time of data collection, we performed a translation/validation process of the questionnaire. The final results are still in process. Before administration to the ID subsample, we decided to make a minor semantic change to the original version of the instrument and add a visual aid (“smiley faces”) so as to improve the poor comprehension observed during the pilot study. Our modified version was approved by the DISQOL Group and entitled Experimental Brazilian Version for Persons with Intellectual Disabilities. The overall score range is 5 to 60, the highest score meaning the highest functionality.

2. “About you” questionnaire: designed to collect sociodemographic data and to assess participants’ perceptions of their health status and disability.

3. WHOQOL-Dis: comprised by (1) the WHOQOL-Bref and (2) the original DISQOL module (13 international items) (Table 1). For the PD subsample we kept the original item set and response scale of WHOQOL-Bref. For the ID subsample, minor semantic changes were made to the WHOQOL-Bref items so as to facilitate comprehension. The response scale was changed to a three-point system, except for items 1 and 27, which were considered anchor questions and continued to be scored on a five-point scale. Furthermore, a visual aid (“smiley faces”) was added. The instrument comprised by the local version of WHOQOL-Bref plus the international DISQOL add-on module was named WHOQOL-Dis-D (for use in persons with PD) and WHOQOL-Dis-ID (for use in persons with ID). Additionally, the five add-on local items (as used in the pilot study) were also included in the scales.

**Table 1:** Factors and items of the DISQOL module (a)

**Factors and items**	
Overall	
	Does your disability have a negative (bad) effect on your day-to-day life?
F1	
	Do you feel that some people treat you unfairly?
	Do you need someone to stand up for you when you have problems?
	Do you worry about what might happen to you in the future? *For example, thinking about not being able to look after yourself, or being a burden to others in the future.*
F2	
	Do you feel in control of your life? *For example, do you feel in charge of your life?*
	Do you make your own choices about your day-to-day life? *For example, where to go, what to do, what to eat.*
	Do you get to make the big decisions in your life? *For example, like deciding where to live, or who to live with, how to spend your money.*
F3	
	Are you satisfied with your ability to communicate with other people? *For example, how you say things or get your point across, the way you understand others, by words or signs.*
	Do you feel that other people accept you?
	Do you feel that other people respect you? For example, do you feel that others value you as a person and listen to what you have to say?
	Are you satisfied with your chances to be involved in social activities? *For example, meeting friends, going out for a meal, going to a party etc.*
	Are you satisfied with your chances to be involved in local activities? *For example, being part of what is happening in your local area or neighbourhood.*
	Do you feel that your dreams, hopes and wishes will happen? *For example, do you feel you will get the chance to do the things you want, or get the things you wish for, in your life?*
Local Module	
	Are you satisfied with the opportunities you have to work?*For example, with the job offers you receive.*
	Are you satisfied with the adaptations of your environment to your limitation?*For example, access ramps, adapted restrooms, elevators, in the case of moving difficulty; signaling in the streets, in the case of visual impairment; sign language interpreters, in the case of hearing impairment.*
	Are you satisfied with the opportunities you have to study?*For example, if you want a school or university to accept you as a student.*
	Are you satisfied with your nutrition?*For example, with the amount and quality of the food you eat.*

(a) DISQOL Module – WHO’s Quality of Life Module for People with Intellectual and Physical Disabilities.

4. Satisfaction with Life Scale (unpublished observations: Hutz CS, Giacomoni CH: Adaptação da Escala de Satisfação de Diener para o Brasil. Porto Alegre: UFRGS; 1998): included for hypothesis validation (convergent validity). It consists of five questions scored on a scale of 1 to 5, so that the lowest possible overall score (poorest satisfaction with life) is 5 and the highest possible score (greatest satisfaction with life) is 25. Cronbach alpha: 0.82 [18].

5. Beck Depression Inventory, version II (BDI-II) [19]: 21-item depression assessment scale translated and validated into Brazilian Portuguese [20]. Included for hypothesis validation (discriminant validity). The score ranges from 0 (zero) to 63 – the highest score meaning the greatest the depressive symptoms. Cronbach alpha: 0.92 [20].

Due to convenience reasons, retesting was restricted to the ID subsample. Because it was usually not possible to know in advance the kind of PD the participant had, such that we could not be prepared in advance, people with hearing impairment had to be able to read Brazilian Portuguese and people with visual impairment had to be willing to have the instruments read by the administrator. In the end of the administration there was a space for the participant to register comments, suggestions, and difficulties on the completion of questionnaires.

### Inclusion and exclusion criteria

**Figure 1:**
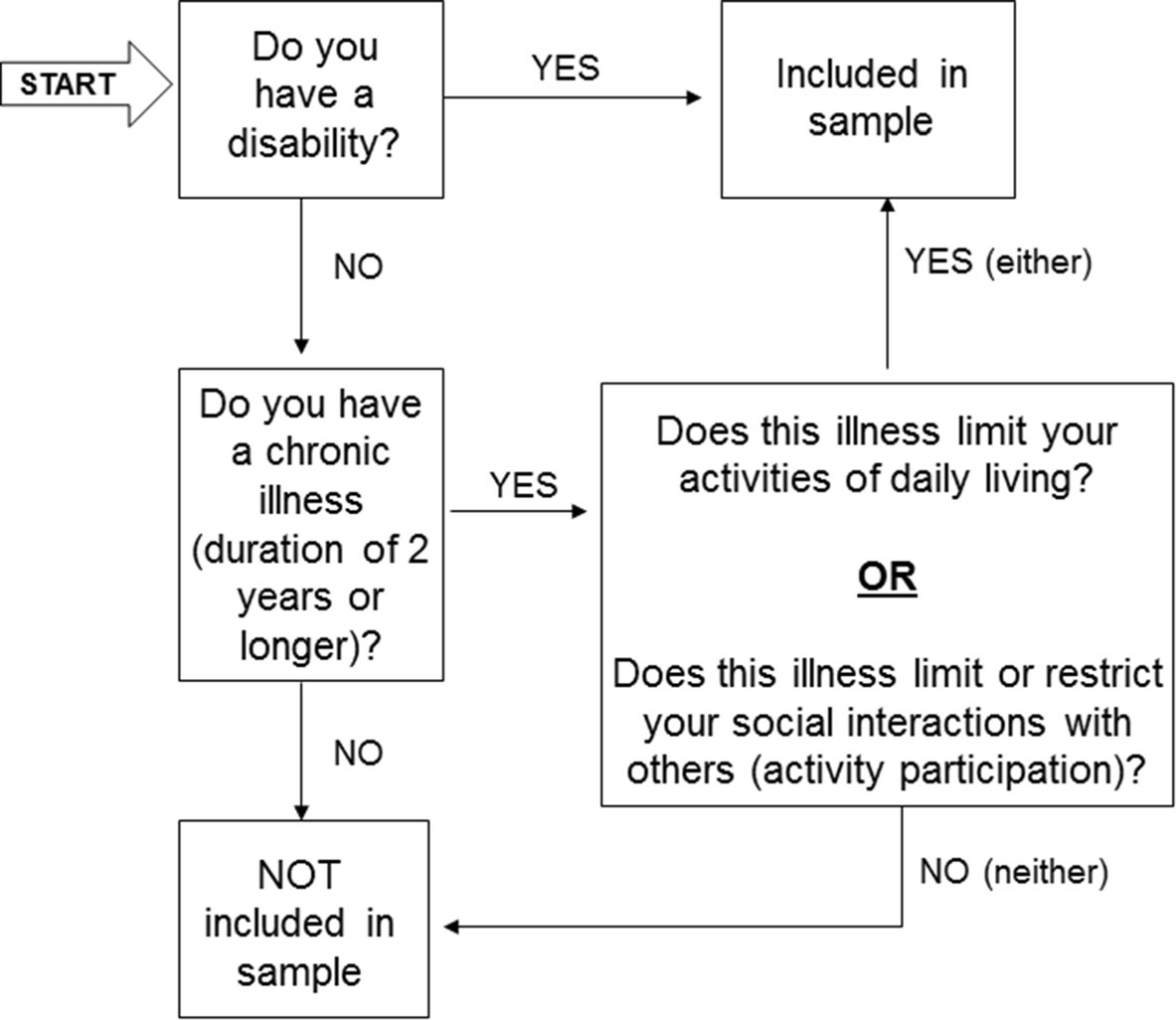
Inclusion flowchart for participants with physical disability.

Because of sampling convenience, all participants were required to attend (or be institutionalized at) a facility specializing in the care of persons with disability, non-governmental organization, school, or health care facility. Only adults (age 18–65) were considered for participation. The WHOQOL Group establishes a number of participants per centre based on extensive experience in studies of this nature. Because this is a cross-cultural, simultaneous and original study, it is difficult to rely on literature or sample calculations. Thus, it was stipulated the goal of 150 people with PD and 150 people with intellectual disabilities for each participating centre [21]. As the concept of disability has not yet been widely disseminated in Brazil, a flowchart was devised and administered to each potential subject with PD prior to inclusion in the sample (Figure 1). ID participants were included only when cared for at a service dealing specifically with the care of persons with ID. In addition, a screening step was used to evaluate the ability of participants to respond to the study questionnaire in an assertive manner, ensuring that only persons with mild ID were included in the sample. Two instruments were used for screening: Test of Acquiescence (adapted from Cummins [22]), designed to determine whether the subject merely tends to agree with the interviewer’s questions (acquiescent responding) or is capable of providing actual answers even to reverse-scored questions; and the Test for Discriminative Competence (adapted from Dalton and McVilly [23]), which seeks to ascertain whether the participant is able to discriminate his/her chosen response on a three-point scale. Participants with ID were excluded from the sample after failure in both screening tests.

### Ethical aspects

The project was approved by the Hospital de Clínicas de Porto Alegre Research Ethics Committee (processes 06–016, 06–017, and 06–021). The wording of the written informed consent (WIC) form stressed the possibility of dropping out of the study at any time if desired. For participants in the ID subsample, WIC was also required from one of the participant’s parents or legal guardians. Participants with ID who were excluded from the study due to failure at screening tests were not told why their participation was being terminated. An intervention protocol was devised for participants with moderate-to-severe depression or a score of 2 or 3 on the suicidal ideation item (question 9) of the BDI-II. Protocol interventions ranged from notifying the participant of the need for in-depth assessment for depression to notification of the care team in participants positive for suicidal ideation. This protocol was the subject of extensive discussions between the investigators and the Hospital de Clínicas de Porto Alegre Research Ethics Committee. All study procedures were carried out in accordance with the standards of the Declaration of Helsinki [24] and participant anonymity and confidentiality were preserved at all times.

### Data analysis

All analyses were based on classical psychometric methods and were conducted independently for the two study samples (PD and ID). Shapiro–Wilk and Kolmogorov–Smirnov tests were used to evaluate the normality of distribution and 95% confidence intervals were calculated. Missing data were replaced with the medians of nearby points. Exploratory factor analyses were carried out using principal component analysis with Varimax rotation and Kaiser normalization.

Evidence of construct validity was based on the responses to the questions “Are you currently ill or in poor health?” and “Do you have a disability [impairment or limitation]?” for the establishment of criterion groups. Yes-or-no answers to the former question resulted in the “perception of health” variable, whereas yes-or-no answers to the latter question resulted in the “perception of disability” variable. Each variable was analyzed in relation to the WHOQOL-Dis domains by means of Student’s *t*-test for independent samples if normally distributed or the Mann–Whitney U test if otherwise. Exploratory factor analysis was performed with both subsamples. Models were also forced into the number of factors present in the cross-cultural solution.

Evidence of criterion validity was also obtained by means of Student’s *t*-test for independent samples (for normally distributed variables) or the Mann–Whitney U test (for variables with a skewed distribution) in relation to the SWLS and BDI-II measure (concurrent validity) and in relation to age, years of study, and disability as expressed by WHODAS II score (discriminant validity).

Cronbach alpha was calculated to obtain evidence of reliability for the instrument scales and subscales. In the ID subsample, test-retest reliability was analyzed by means of the intraclass correlation coefficient (ICC, average measures, two-way-random) and Student’s *t*-test for paired samples (domain averages).

All analyses were carried out in the Statistical Package for the Social Sciences^TM^ (SPSS) version 18.0.

## Results

The PD subsample comprised participants with a variety of health conditions, the most common being visual impairment (15.4%), hearing impairment (6.8%), and stroke sequelae (2.5%). A total of 162 (98 females) people with PD participated in study, with a mean age of 45,48 (SD 12,76). In the subsample of people with ID, the number of participants reached 156 (55 females), mean age of 30,53 (SD 9,42). Overall, the missing data rates were 0.64% for the PD subsample and 0.42% for the ID subsample. Among the PD group, the highest rate of missing data (3.7%) was found in local item 42 “Are you satisfied with your opportunities for study? For example, if you would like a school or university to take you on as a student”, whereas among the ID group the highest rate of missing data (4.48%) was found in local item 41 “Are you satisfied with how your environment is adapted to your limitation? For example: wheelchair ramps, accessible toilets, elevators (if you have mobility limitations); street and road signage (if you have visual impairment); sign language interpreters (if you have hearing impairment)”. As to difficulties in the completion of the questionnaires (qualitative data), no participant declared problems as to the administration style.

Exploratory factor analysis (Tables 2 and 3) revealed slightly different models from the original cross-cultural model. In Tables 4 and 5 the results for models forced into three factors to match the cross-cultural model are shown. This solution revealed a different model in terms of factor positioning, with factor 2 (F2) being the most important in explaining variance.

**Table 2:** Exploratory factor analysis: physical disability subsample (n = 162)

**Item**	**Component**				**Original factor in DISQOL international**
	**1**	**2**	**3**	**4**	
32 - Choice	**0.842**	0.231	−0.02	0.097	F2
33 - Autonomy	**0.769**	−0.06	0.206	0.04	F2
31 - Control	**0.736**	0.193	0.017	0.289	F2
34 - Communication	**0.431**	0.276	0.38	−0.157	F3
37 - Interaction	0.154	**0.832**	0.156	0.184	F3
38 - Inclusion	0.152	**0.793**	0.169	0.079	F3
39 - Potential	0.053	**0.676**	0.081	−0.123	F3
36 - Respect	0.101	0.097	**0.841**	0.008	F3
35 - Acceptance	0.188	0.255	**0.766**	−0.049	F3
28 - Discrimination	−0.08	0.064	**0.642**	0.453	F1
30 - Future prospects	0.09	−0.01	−0.04	**0.745**	F1
29 - Advocacy	0.138	0.047	0.088	**0.701**	F1

KMO: 0.725.

Bartlett’s test: p < 0.001.

Explained variance: 63.14%.

**Table 3:** Exploratory factor analysis: intellectual disability subsample (n = 156)

**Item**	**Component**				**Original factor in DISQOL international**
	**1**	**2**	**3**	**4**	
36 - Respect	**0,805**	0,053	0,166	−0,021	F3
35 - Acceptance	**0,738**	0,145	0,188	−0,142	F3
28 - Discrimination	**0,617**	−0,017	−0,139	0,552	F1
38 - Inclusion	0,003	**0,833**	0,04	0,059	F3
37 - Interaction	0,124	**0,783**	0,172	0,064	F3
34 - Communication	0,432	**0,441**	0,234	−0,022	F3
33 - Autonomy	0,09	0,001	**0,777**	0,011	F2
32 - Choice	0,178	0,102	**0,768**	0,157	F2
31 - Control	0,08	0,227	**0,575**	−0,023	F2
29 - Advocacy	−0,077	−0,059	0,195	**0,757**	F1
30 - Future prospects	0,041	0,323	0,032	**0,628**	F1
39 - Potential	0,248	0,385	0,137	**−0,43**	F3

KMO: 0.688.

Bartlett’s test: p < 0.001.

Explained variance: 58.277%.

**Table 4:** Exploratory factor analysis, model forced into three factors: physical disability subsample (n = 162)

**Item**	**Component**			**Original factor in DISQOL international**
	**1**	**2**	**3**	
38 - Inclusion	**0.721**	0.156	0.139	F3
37 - Interaction	**0.709**	0.214	0.177	F3
39 - Potential	**0.649**	−0.02	−0.01	F3
34 - Communication	**0.517**	0.241	0.169	F3
31 - Control	0.253	**0.772**	0.02	F2
32 - Choice	0.377	**0.767**	−0.12	F2
33 - Autonomy	0.201	**0.647**	0.062	F2
29 - Advocacy	−0.168	**0.471**	0.387	F1
30 - Future prospects	−0.28	**0.467**	0.31	F1
28 - Discrimination	0.044	0.093	**0.782**	F1
36 - Respect	0.35	−0.01	**0.707**	F3
35 - Acceptance	**0.505**	0.046	**0.596**	F3

KMO: 0.725.

Bartlett’s test: p < 0.001.

Explained variance: 53.641%.

**Table 5:** Exploratory factor analysis, model forced into three factors: intellectual disability subsample (n = 156)

**Item**	**Component**			**Original factor in DISQOL international**
	**1**	**2**	**3**	
37 - Interaction	**0.767**	0.067	0.035	F3
38 - Inclusion	**0.749**	−0.09	0.014	F3
34 - Communication	**0.491**	0.42	−0.033	F3
31 - Control	**0.473**	0.202	0.037	F2
32 - Choice	**0.451**	0.361	0.245	F2
33 - Autonomy	**0.371**	0.293	0.111	F2
36 - Respect	0.11	**0.807**	−0.032	F3
35 - Acceptance	0.204	**0.738**	−0.152	F3
28 - Discrimination	−0.1	**0.551**	0.505	F1
29 - Advocacy	0.033	−0.019	**0.781**	F1
30 - Future prospects	0.29	0.003	**0.604**	F1
39 - Potential	**0.403**	0.228	**−0.439**	F3

KMO: 0.688.

Bartlett’s test: p < 0.001.

Explained variance: 48.228%.

The results of internal consistency analysis of models constrained to three factors are shown in Table 6. Cronbach alpha was satisfactory [25] across practically all domains and factors in the PD subsample; values <0.70 were found only in the Social domain and in Factor 1 (both of them containing only three items). In the ID subsample, most factors or domains had coefficients higher than 0.70, but four subscales exhibited less satisfactory performance.

**Table 6:** Evidence of internal consistency

**Domain/factor**	**Cronbach alpha**		**No. of items**
	**PD (n = 162)**	**ID (n = 156)**	
Domain 1: Physical^(a)^	.839	.566	7
Domain 2: Psychological^(a)^	.745	.565	6
Domain 3: Social^(a)^	.663	.423	3
Domain 4: Environment^(a)^	.722	.673	8
WHOQOL-Bref	.898	.840	26
Factor 1^(b)^	.441	.493	3
Factor 2^(b)^	.757	.588	3
Factor 3^(b)^	.746	.685	6
Factor 4^(c)^	.760	.690	12
DISQOL module	.771	.692	13
WHOQOL-Dis^(d)^	.910	.858	37

^(a)^WHOQOL-Bref. ^(b)^DISQOL module: factors consistent with those identified in the original cross-cultural project (global sample). ^(c)^Single factor suggested by the coordinating centre (POWER et al., [12]) as an alternative solution to the DISQOL module. ^(d)^WHOQOL-Bref + DISQOL module.

Regarding concurrent validity, both WHOQOL-Bref and the DISQOL module discriminated groups stratified by level of depression in the PD and ID subsamples alike (Tables 7 and 8). Evidence of construct validity for “perception of health” in the PD subsample was obtained in all factors except in Factor 1. In the ID subsample, discriminative capacity between these criterion groups (healthy and not healthy) was inconsistent (Tables 9 and 10).

**Table 7:** Discriminant validity of WHOQOL-DIS for people with physical disabilities

**Factor/domain**	**Age (years)**				**Disability (WHODAS-II)**				**Years of study**			
	**18-40 (n = 54)**	**41-65 (n = 108)**	**df = 160**		**12-26 (n = 83)**	**27-55 (n = 79)**	**df = 160**		**0 -7 (n = 82)**	**8 -21 (n = 74)**	**df = 154**	
	**Mean (SD)**	**Mean (SD)**	***t***	**d**	**Mean (SD)**	**Mean (SD)**	***t***	**d**	**Mean (SD)**	**Mean (SD)**	***t***	**d**
Physical^(a)^	63,69 (19,27)	44,35 (19)	6,08**	0,433	61,83 (16,71)	39,2 (19)	8,06**	0,537	43,21 (19,58)	59,51 (19,21)	−5,24**	0,389
Psychological^(a)^	67,44 (18,12)	62,19 (15,55)	1,91*	0,149	70,63 (11,58)	56,91 (18,12)	5,71**	0,411	59,81 (17,41)	68,75 (14,7)	−3,45**	0,268
Social^(a)^	72,69 (21,62)	63,43 (18,44)	2,84*	0,219	71,69 (18,36)	61,08 (20,27)	3,49**	0,266	62,6 (19,21)	71,51 (19,85)	−2,85*	0,224
Environment^(a)^	55,32 (14,17)	55,03 (16,16)	0,11	0,009	59,34 (13,32)	50,71 (16,41)	3,68**	0,279	51,03 (15,37)	60,09 (14,19)	−3,81**	0,293
Overall^(a)^	64,58 (20,71)	51,5 (21,26)	3,72**	0,282	65,06 (18,5)	46,2 (21,12)	6,05**	0,431	49,09 (21,85)	63,85 (19,58)	−4,43**	0,336
Factor1^(b)^	64,81 (20,07)	63,58 (22,75)	0,34	0,027	69,38 (19,7)	58,33 (22,65)	3,32**	0,254	61,79 (22,98)	66,67 (19,99)	−1,42	0,114
Factor2^(b)^	71,14 (23,27)	71,45 (24,31)	−0,08	0,006	74,7 (19,76)	**67,83 (27,27)**	**−1,22**	**−0,096**	70,02 (25,76)	73,87 (21,07)	−1,03	0,083
Factor3^(b)^	69,06 (17,67)	66,09 (18,33)	0,98	0,077	70,88 (15,23)	63,08 (20,05)	2,78*	0,215	66,67 (19,93)	68,47 (15,61)	−0,63	0,051
Overall^(b)^	14,93 (6,91)	11,92 (7,62)	2,44*	0,189	16,04 (6,41)	9,65 (7,2)	5,97**	0,427	11,66 (7,84)	14,36 (6,84)	−2,28*	0,181
DISQOL Module	68,52 (15,38)	66,8 (15,09)	0,68	0,054	71,46 (12,83)	63,08 (16,27)	3,63**	0,276	66,29 (15,39)	69,37 (14,36)	−1,29	0,103
Local Module	55,56 (21,57)	45,31 (19,2)	3,07**	0,236	53,84 (20,05)	43,35 (19,77)	3,35**	0,256	44,66 (20,3)	54,14 (20,04)	−2,93*	0,230

^(a)^WHOQOL-Bref. ^(b)^DISQOL Module: factors congruent to the ones found in the cross-cultural project (global sample).

Levels of significance: *p < 0.05; **p ≤ 0.001. In bold: Mann–Whitney statistics for non-normal distributions. df = degrees of freedom; d = effect size.

**Table 8:** Discriminant validity of WHOQOL-DIS for people with intellectual disabilities

**Factor/domain**	**Age (years)**				**Disability (WHODAS-II)**			
	**18-40 (n = 130)**	**41-57 (n = 26)**	**df = 154**		**12-18 (n = 82)**	**19-51 (n = 74)**	**df = 154**	
	**Mean (SD)**	**Mean (SD)**	***t***	**d**	**Mean (SD)**	**Mean (SD)**	***t***	**d**
Physical^(a)^	52,66 (9,13)	55,36 (7,37)	−1,41*	0,113	56,49 (7,36)	49,37 (8,99)	5,37**	0,397
Psychological^(a)^	49,13 (8,39)	50,64 (8,31)	−0,84	0,068	51,83 (6,81)	46,68 (9,12)	3,96**	0,304
Social^(a)^	40,19 (11,71)	38,46 (9,74)	0,71	0,057	44 (9,1)	35,36 (12,01)	5,02**	0,375
Environment^(a)^	38,15 (9,32)	40,14 (10,33)	−0,98	0,079	41,73 (7)	34,88 (10,58)	4,71**	0,355
Overall^(a)^	80,96 (23,77)	78,37 (23,06)	0,51	0,041	86,89 (17,88)	73,48 (27,05)	3,61**	0,279
Factor 1^(b)^	76,86 (14,97)	75,32 (15,36)	0,48	0,039	79,88 (14,87)	72,97 (14,39)	2,94*	0,231
Factor 2^(b)^	31,99 (15,38)	31,41 (13,4)	0,18	0,015	34,65 (14,01)	28,83 (15,62)	2,46*	0,194
Factor 3^(b)^	39,62 (10,3)	39,74 (10,82)	−0,06	0,005	42,99 (7,72)	35,92 (11,62)	4,42**	0,336
Overall^(b)^	15 (9,42)	16,59 (8,09)	−0,8	0,064	17,45 (8,02)	12,84 (9,86)	3,19**	0,249
DISQOL Module	47,02 (8,78)	46,55 (10,05)	0,24	0,019	50,13 (8,06)	43,41 (8,64)	5,02**	0,375
Local Module	38,56 (10,36)	38,7 (11,04)	−0,06	0,005	41,54 (8,53)	35,3 (11,4)	3,84**	0,296

^(a)^WHOQOL-Bref. ^(b)^DISQOL Module: factors congruent to the ones found in the cross-cultural project (global sample).

Levels of significance: *p < 0.05; **p ≤ 0.001. df = degrees of freedom; d = effect size.

**Table 9:** Construct and concurrent validity of WHOQOL-DIS for people with physical disabilities

**Factor/domain**	**Are you currently ill or in poor health?**				**Do you have a disability [impairment or limitation]?**				**Depression (BDI-II) (stratified by quantils)**					**Satisfaction with life (SWLS) (stratified by quantils)**		
	**Yes (n = 113)**	**No (n = 49)**	**df = 160**		**Yes (n = 155)**	**No (n = 7)**	**df = 160**		**≤ 11 (n = 81)**	**≥ 12 (n = 81)**	**df = 160**		**≤ 23 (n = 80)**	**≥ 24 (n = 82)**	**df = 160**	
	**Mean (SD)**	**Mean (SD)**	***t***	**d**	**Mean (SD)**	**Mean (SD)**	***t***	**d**	**Mean (SD)**	**Mean (SD)**	***t***	**d**	**Mean (SD)**	**Mean (SD)**	***t***	**d**
Physical^(a)^	42,19 (17,44)	70,63 (14,49)	−10,01**	0,62	50,41 (21,07)	59,18 (22,1)	−1,07	0,08	61,29 (17,34)	40,3 (19,34)	7,27**	0,50	45,22 (19,82)	56,23 (21,04)	−3,42**	0,26
Psychological^(a)^	60,67 (18,81)	71,51 (13,8)	−4,0**	0,30	63,71 (16,79)	69,05 (10,45)	−0,83	0,07	73,46 (11,89)	54,42 (15,64)	8,91**	0,58	57,19 (17,67)	70,53 (12,37)	−5,56**	0,40
Social^(a)^	63,42 (18,81)	73,64 (20,93)	−3,07**	0,24	66,4 (19,89)	69,05 (23,43)	−0,34	0,03	73,77 (17,83)	59,26 (19,45)	4,95**	0,36	58,75 (20,41)	74,09 (16,41)	−5,28**	0,39
Environment^(a)^	53,43 (15,88)	59,06 (13,86)	−2,15*	0,17	54,68 (15,39)	65,18 (14,92)	−1,77*	0,14	63,58 (11,6)	46,68 (14,25)	8,28**	0,55	49,73 (15,82)	60,40 (13,23)	−4,66**	0,35
Overall^(a)^	48,56 (20,37)	72,7 (15,02)	−8,39**	0,55	55,89 (21,9)	55,36 (23,78)	0,06	0,00	63,73 (19,12)	47,99 (21,78)	4,89**	0,36	47,97 (21,55)	63,57 (19,46)	−4,84**	0,36
Factor1^(b)^	62,54 (22,8)	67,35 (19,23)	−1,29	0,10	63,23 (21,69)	80,95 (19,07)	−2,12*	0,17	72,33 (19,27)	55,66 (21,16)	5,24**	0,38	**61,25 (20,81)**	**66,67 (22,6)**	−1,68*	**−0,13**
Factor2^(b)^	68,88 (25,44)	77,04 (18,91)	−2,26*	0,18	70,5 (24,01)	88,09 (13,49)	−1,91*	0,15	79,01 (17,44)	63,68 (29,95)	4,3**	0,32	65,83 (25,87)	76,73 (20,57)	−2,96*	0,23
Factor3^(b)^	65,41 (19,17)	70,92 (14,87)	−1,98*	0,15	66,34 (18,01)	83,33 (12,5)	−2,46*	0,19	74,18 (14,66)	59,98 (18,53)	5,41**	0,39	61,97 (19,66)	72,05 (14,98)	−3,66**	0,28
Overall^(b)^	10,84 (7,04)	17,73 (6,3)	−5,9**	0,42	12,74 (7,47)	16,96 (7,83)	−1,46	0,11	16,13 (6,08)	9,72 (7,46)	5,99**	0,43	10,47 (7,02)	15,32 (7,23)	−4,33**	0,32
DISQOL Module	65,56 (15,96)	71,56 (12,28)	−2,6*	0,20	66,63 (14,92)	83,93 (10,94)	−3,03**	0,23	74,93 (12,18)	59,83 (14,09)	7,29**	0,50	62,76 (15,36)	71,87 (13,6)	−4,0**	0,30
Local Module	44,3 (20,02)	58,93 (18,09)	−4,39**	0,33	47,94 (20,3)	66,07 (19,38)	−2,31*	0,18	55,48 (18,76)	41,98 (20,11)	4,42**	0,33	46,64 (18,92)	50,76 (21,92)	−1,28	0,10

^(a)^WHOQOL-Bref. ^(b)^DISQOL Module: factors congruent to the ones found in the cross-cultural project (global sample).

Levels of significance: *p < 0.05; **p ≤ 0.001. In bold: Mann–Whitney statistics for non-normal distributions. df = degrees of freedom; d = effect size.

**Table 10:** Construct and concurrent validity of WHOQOL-DIS for people with intellectual disabilities

**Factor/domain**	**Do you have a disability [impairment or limitation]?**				**Depression (BDI-II) (stratified by quantils)**				**Satisfaction with life (SWLS) (stratified by quantils)**			
	**Yes (n = 94)**	**No (n = 61)**	**df = 153**		**≤ 5 (n = 72)**	**≥ 6 (n = 83)**	**df = 153**		**≤ 30 (n = 73)**	**≥ 31 (n = 83)**	**df = 154**	
	**Mean (SD)**	**Mean (SD)**	***t***	**d**	**Mean (SD)**	**Mean (SD)**	***t***	**d**	**Mean (SD)**	**Mean (SD)**	***t***	**d**
Physical^(a)^	52,09 (8,89)	54,81 (8,75)	−1,87*	0,15	56,35 (6,89)	50,3 (9,55)	4,56**	0,35	50,49 (10,16)	55,42 (6,88)	−3,5*	0,27
Psychological^(a)^	48,98 (8,35)	50,14 (8,44)	−0,84	0,07	52,78 (5,89)	46,44 (9, 14)	5,2**	0,39	47,09 (9,36)	51,41 (6,83)	−3,25**	0,25
Social^(a)^	39,89 (11,39)	40,44 (10,85)	−0,3	0,02	43,29 (9,65)	37,05 (12,09)	3,57**	0,28	35,62 (12,29)	43,67 (9,07)	−4,61**	0,35
Environment^(a)^	38 (9,27)	39,34 (9,87)	−0,86	0,07	40,28 (8,61)	36,86 (10,01)	2,26*	0,18	34,59 (10,3)	41,91 (7,19)	−5,08**	0,38
Overall^(a)^	77,79 (24,26)	85,25 (21,83)	−1,94*	0,15	84,72 (20,15)	76,96 (25,93)	2,09*	0,17	72,43 (27,56)	87,65 (16,62)	−4,11**	0,31
Factor1^(b)^	74,02 (14,86)	81,01 (14,04)	−2,92*	0,23	80,67 (14,59)	73,29 (14,54)	3,15**	0,25	73,74 (13,3)	79,12 (16,01)	−2,26*	0,18
Factor2^(b)^	31,91 (15,25)	31,97 (14,92)	−0,02	0,00	34,72 (13,92)	29,22 (15,53)	2,31*	0,18	27,28 (14,52)	35,94 (14,37)	−3,74**	0,29
Factor3^(b)^	39,01 (10,2)	40,85 (10,48)	−1,08	0,09	42,07 (8,04)	37,4 (11,62)	2,94*	0,23	35,9 (11,47)	42,92 (7,99)	−4,38**	0,33
Overall^(b)^	13,36 (8,91)	18,34 (8,9)	−3,4**	0,27	16,67 (8,39)	14,01 (9,79)	1,8*	0,14	13,18 (9,05)	17,09 (9)	−2,7*	0,21
DISQOL Module	45,99 (8,83)	48,67 (8,85)	−1,84*	0,15	49,88 (8,04)	44,33 (9,01)	4,02**	0,31	43,21 (8,66)	50,23 (7,93)	−5,28**	0,39
Local Module	38,7 (10,5)	38,63 (10,36)	0,04	0,00	40,71 (9,26)	36,59 (11,05)	2,49*	0,20	35,36 (11,33)	41,42 (8,71)	−3,7**	0,29

^(a)^WHOQOL-Bref. ^(b)^DISQOL Module: factors congruent to the ones found in the cross-cultural project (global sample).

Levels of significance: *p < 0.05; **p ≤ 0.001. df = degrees of freedom; d = effect size.

Of the 96 participants with ID who completed retesting, 27 were excluded from the retest reliability analysis due to occurrence of significant life events (positive or negative) during the interval between test and retest. The results of this analysis are shown in Table 11.

**Table 11:** Test-retest reliability among participants with intellectual disability

**Factor/domain (t1-t2) (c)**	***t-*test for paired samples (df = 68)**		**Intraclass correlation coefficient (ICC)**
	**t**	**d**	**F (95% CI)**
Physical^(a)^	−0.05	0.01	0.65** (0.43-0.78)
Psychological^(a)^	−033	0.97	0.57** (0.3-0.73)
Social^(a)^	−1.52	0.18	0.46* (0.13-0.67)
Environment^(a)^	−1.27	0.15	0.64** (0.42-0.78)
Overall^(a)^	0.35	0.04	0.48* (0.16-0.68)
Factor 1^(b)^	−0.29	0.04	0.48* (0.16-0.68)
Factor 2^(b)^	−1.13	0.14	0.58** (0.316-0.74)
Factor 3^(b)^	−0.46	0.06	0.72** (0.54-0.82)
Overall^(b)^	−34.17**	0.97	0.41* (0.05-0.64)
DISQOL module	−1.04	0.13	0.74** (0.58-0.84)
Local module	−1.09	0.13	0.36* (−0.03-0.6)

^(a)^WHOQOL-Bref. ^(b)^DISQOL module: factors consistent with those identified in the original cross-cultural project (global sample).

Significance level: *p < 0.05; **p ≤ 0.001 (two-tailed). (c) Mean interval: 28 ± 13.58 days. df = degrees of freedom; d = effect size.

## Discussion

Analysis of item clustering on exploratory factor analysis showed a trend toward the same factors found in the original factor analysis of the instrument [12], although a reverse ranking of factors in terms of explained variance was observed. The initial objective of the WHOQOL-Dis project was to develop cross-cultural instruments to allow intercultural research. Hence, we understand that the use of the Brazilian Portuguese versions of this instrument with the underlying factor structure generated during the original cross-cultural project will not be deleterious in any way. Furthermore, we believe that exploratory investigation of new models after the administration of the instrument to different samples will contribute to the validation of the best-fitting model for use in Brazilian populations.

Analysis of results, in turn, revealed discriminative competence for the “disability” variable as defined by WHODAS-II [17] in both versions of the instrument (PD and ID). Conversely, there was no discriminative competence for the “age” variable in the ID sample, which may suggest this is not a determinant variable of QoL among persons with ID. One could speculate that the inclusion of older adults (age >65 years) with ID in the sample might have produced different findings. The predominance of significant between-group differences in WHOQOL-Bref domains, but not in DISQOL domains, for the variable “years of study” may suggest that the impact of disability on overall QoL is not determined by educational achievement. This variable was only analyzed in the PD group.

The fact that F1 did not show evidence of construct validity for “perception of health” in the PD subsample may suggest that perceptions of need for advocacy and protection in the future among persons with PD are not mediated by the perception of illness. The lack of discriminative capacity between the healthy and not healthy ID groups may be attributable to our failure to select actually “healthy” and “not healthy” participants with ID. In other words, our participants with ID may have positively replied to the question “Are you currently ill or in poor health” due to the presence of mild, acute illness (flu, minor viral infections, etc.). Also no discriminative capacity was found between the criterion groups generated by the question “Do you have a disability (impairment or limitation)?” in the ID subsample. This suggests that whether people with ID are aware of their disability has no bearing on their QoL. In the PD subsample, a few participants claimed they did not have a disability (n = 7), which has a negative effect on the statistical power of our analyses. At any rate, this assessment showed markedly superior performance for the DISQOL module as compared with WHOQOL-Bref for between-group discrimination, which may suggest that the module is competent to discriminate groups only when items sensitive to people with disabilities are present.

As to concurrent validity, “level of depression” has often been described as a concurrent variable to QoL, as these two variables are often observed to exhibit strong negative correlations with one another [26,27]. Conversely, the “satisfaction with life” variable [18] correlates positively with QoL. Strong correlations between these two variables have been described in people with ID [28] and PD [29]. In both subsamples of the present study, WHOQOL-Bref and the DISQOL module discriminated between groups stratified by satisfaction with life, whereas the local module of DISQOL showed no such discriminative competence in the PD subsample. This may be explained by the fact that local items, included as the “Local Module” for purposes of analysis, were not designed to constitute a subscale but rather to account for important topics that arose during local focus group discussions and were not covered by the original cross-cultural items. To facilitate cross-cultural use of the developed scales, the loading of local questions on other domains was not explored; this may be an avenue for future research.

Reliability, as expressed by Cronbach alpha, was satisfactory in the PD subsample and less so in the ID subsample. This is consistent with the findings of Streiner [30], who maintains that coefficients <0.70 are to be expected when analyzing scales with few items. Cronbach alpha is highly influenced by size and number of items in each factor [30]. Reliability results were better when WHOQOL-Bref items were clustered and better still when WHOQOL-Bref was analyzed jointly with the DISQOL module, which reinforces the WHO orientation that specific modules should always be administered jointly with their original instruments [12].

Analysis of test-retest reliability, carried out in the ID subsample, revealed highly significant intraclass correlation coefficients, consistent with the absence of significance on Student’s *t*-test, which suggests satisfactory reliability. One exception was the Overall item of the DISQOL module, which may suggest that isolated items may not perform well in this population.

The possibility of developing a measurement instrument for persons with ID has been widely discussed and called into question [12]. Such discussions have centred on whether people with ID are capable of assessing a subjective construct such as QoL or whether research should focus on the development of QoL assessment instruments for completion by proxy. The findings of the present study point to satisfactory validity and reliability for an assessment of QoL among participants with ID, which suggests that persons with mild cognitive impairment are capable of reporting their perceptions in a valid and reliable way. Nevertheless, further research is still required to confirm these findings in different samples. It bears noting that the DISQO Project also sought to triangulate all information obtained from ID participants with data provided by their caregivers and relatives. These findings will be the subject of a future publication.

Regarding the limitations of this study, a brief discussion on the Portuguese word *incapacidade* (the term used in this version of the instrument for disability, literally “dis-capacity”) and participants’ understanding thereof is in order. The English word disability poses a challenge for translation into Portuguese. The translation of this term as *incapacidade* prompted spontaneous criticism from several participants, as reported qualitatively by the investigators involved in data collection. This perception may have acted as a confounding variable; its impact as such on study outcomes is difficult to estimate.

The use of different methods of administration should also be taken as a study limitation. Otherwise, the greatest limitations of this study concern the convenience sampling strategy and the sample size, which limit generalization of findings to other samples. Therefore, we hope that future studies can provide additional evidence of the validity of the instruments developed and reported herein.

## Conclusions

The present study sought to present evidence of validity and reliability of a cross-cultural instrument developed for the assessment of QoL among people with disabilities, as defined by WHO [1]. Analysis of results demonstrated that the DISQOL-D and DISQOL-ID modules (designed for persons with PD and ID respectively) constitute a measurement option for the assessment of QoL of people with PD and ID. It is recommended that the modules always be administered jointly with WHOQOL-Bref, so that general and specific aspects of QoL can be captured. One indirect contribution of this project lies in its empirical demonstration that reliable information may be generated from the use of a self-report questionnaire by participants with mild intellectual impairment, even for the assessment of such an inherently subjective construct as QoL.

Pursuant to the WHOs policy on access to health information, the study instruments are available from the WHOQOL-Brasil Group website. Future research designed to investigate the impact of psychosocial variables on QoL among persons with disabilities would contribute greatly to the collection of information to serve as inputs for the development of public policies mindful of the true needs of this population.

## Abbreviations

BDI-II: Beck depression inventory, version II; DISQOL: Disability – quality of life; ICF: International classification of functioning, disability and health; ID: Intellectual disability; PD: Physical disability; QoL: Quality of life; SPSS: Statistical package for the social sciences; SWLS: Satisfaction with life scale; WHO: World Health Organization; WHODAS-II: World Health Organization Disability Assessment Schedule II; WHOQOL-Bref: World Health Organization Quality of Life Instrument – Abbreviate 26-item Scale; WHOQOL-Dis: World Health Organization Quality of Life [Instrument] for People with Intellectual and Physical Disabilities; WHOQOL-Dis-D: World Health Organization Quality of Life Instrument for People with Physical Disabilities; WHOQOL-Dis-ID: World Health Organization Quality of Life Instrument for People with Intellectual Disabilities; WIC: Written Informed Consent.

## Competing interests

The authors declare that they have no competing interests.

## Authors’ contributions

JB collected the data (along with MPAF), performed the data analysis and interpretation and wrote the first draft of the article. MPAF and GPW contributed to the conception and design of the study and to the data analysis and interpretation. MA contributed to the analysis and interpretation. TSP contributed to literature review and to the elaboration of the manuscript. All authors read and approved the final manuscript.

## Pre-publication history

The pre-publication history for this paper can be accessed here:

http://www.biomedcentral.com/1471-2458/14/538/prepub
